# Modeling and Performance Improvement of the Constant Power Regulator Systems in Variable Displacement Axial Piston Pump

**DOI:** 10.1155/2013/738260

**Published:** 2013-10-24

**Authors:** Sung Hwan Park, Ji Min Lee, Jong Shik Kim

**Affiliations:** School of Mechanical Engineering, Pusan National University, Jangjeon-dong, Geumjeong-gu, Busan 609-732, Republic of Korea

## Abstract

An irregular performance of a mechanical-type constant power regulator is considered. In order to find the cause of an irregular discharge flow at the cut-off pressure area, modeling and numerical simulations are performed to observe dynamic behavior of internal parts of the constant power regulator system for a swashplate-type axial piston pump. The commercial numerical simulation software AMESim is applied to model the mechanical-type regulator with hydraulic pump and simulate the performance of it. The validity of the simulation model of the constant power regulator system is verified by comparing simulation results with experiments. In order to find the cause of the irregular performance of the mechanical-type constant power regulator system, the behavior of main components such as the spool, sleeve, and counterbalance piston is investigated using computer simulation. The shape modification of the counterbalance piston is proposed to improve the undesirable performance of the mechanical-type constant power regulator. The performance improvement is verified by computer simulation using AMESim software.

## 1. Introduction

The pressure regulators of swashplate-type variable displacement axial piston pumps (VDAPP) control the swivel angle, which changes the amount of flow rate to hydraulic circuits. The pressure regulator is operating in accordance with the dynamic response of the discharge pressure, and it supplies pilot flow rate to the control piston which regulates the swivel angle of swashplate. The pressure regulator is mainly divided into the three types depending on the operating method, that is, a flat cut-off type, a differential cut-off type, and a constant power type.

The pressure regulators are usually used to save energy of hydraulic systems in the industrial field. As the hydraulic power unit used for movable equipment has increased, the pressure regulators have been applied in such systems in order to protect prime mover. Most movable hydraulic power unit consist of motor, pumps and reservoir (MPR). An overload of the pump can cause damage to the electric motor and its circuits under a variety of load conditions. To avoid these problems, power regulation of the pump is needed in order to respond to wide varieties of loads without exceeding the maximum power range of the prime mover. In this study, we applied the constant power regulator to the VDAPP so that the angle of the swashplate is automatically decreased according to an increase of the load pressure.

Recently, electronic regulators have been studied and commercialized [1–4]. However, the mechanical regulators are mainly applied in the industrial field because a proportional reducing pressure valve which is used as main part of the electronic regulator has relatively poor durability than mechanical regulator. In recently developed hydraulic regulator systems, both the electrical and mechanical regulators are applied to hydraulic regulator system. In those hydraulic regulator systems, the mechanical regulator is used as emergency equipment so that it only works when the electronic regulator fails. Due to the relatively exceptional durability, the mechanical regulator system is especially adopted to construction equipment and combat vehicles, which are used for long periods in poor conditions. 

On the other hand, the main problem for the development of a mechanical regulator is in the parameter selection of each component. The undesirable performance characteristics such as irregular discharge flow in the constant power area and cut-off area can occur with improper design parameter selection. In order to verify the causes of the undesirable performance, the behavior of an internal system must be analyzed precisely.

In this study, a new method to find out the cause of poor performance of the mechanical regulator system with VDAPP by using the commercial simulation software AMESim (Advanced Modeling Environment for Simulation of Engineering System, version 4.2, LMS, France) is proposed. The design parameters of each component can be applied to a nonlinear virtual model which is based on theoretical analysis by using AMESim software [5, 6]. This approach is used to analyze the internal motion of the spool and sleeve in the regulator which cannot be measured in a real system. In order to verify the accuracy of the suggested model, this simulation result is compared with experimental output in order to validate the simulation.

In Section 2, we present the structure and operating principle of a constant power regulator. A mathematical analysis for the AMESim model of a swash plate VDAPP is introduced in Section 3. In Section 4, we compare the simulation results with the experimental output to validate the simulation model. Then, the shape modification of the counterbalance piston is proposed and the effect of the improvement is verified by computer simulation. Our conclusions are given in Section 5. 

## 2. The Principle of Constant Power Regulator Operation

### 2.1. Structure of a Constant Power Regulator

**Figure 1:**
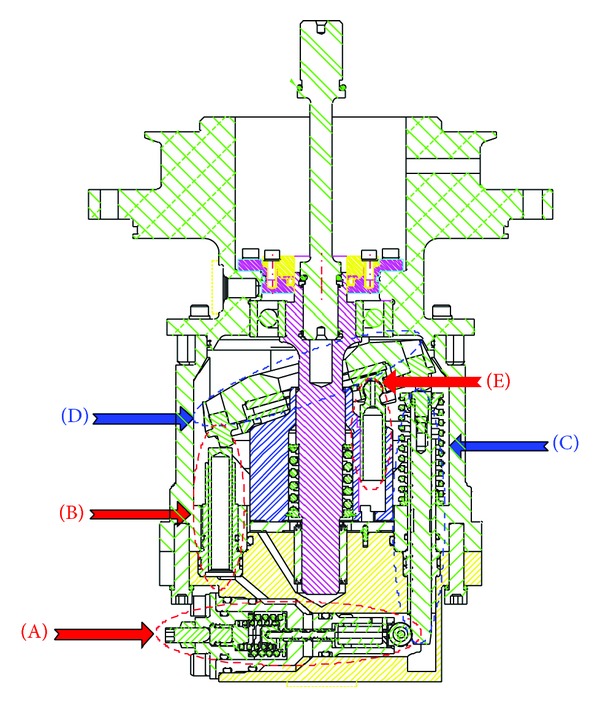
Sectional view of the VDAPP with the mechanical regulator.

**Figure 2:**
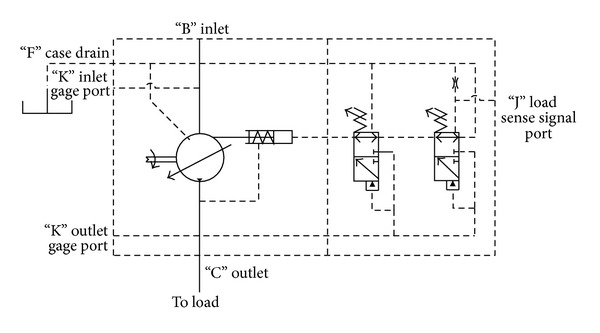
Hydraulic circuit of the mechanical regulator of the VDAPP.

**Figure 3:**
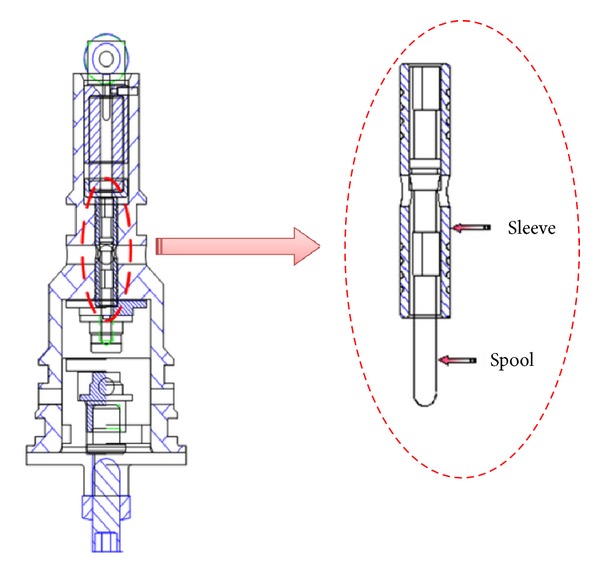
Regulator assembly of constant power regulator.

**Figure 4:**
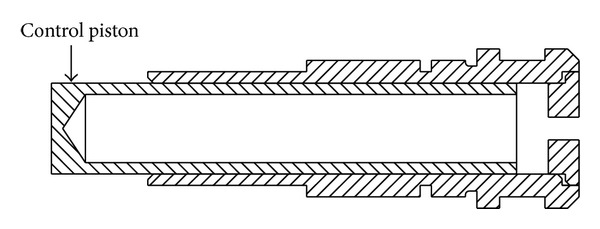
Sectional view of control cylinder assembly.

**Figure 5:**
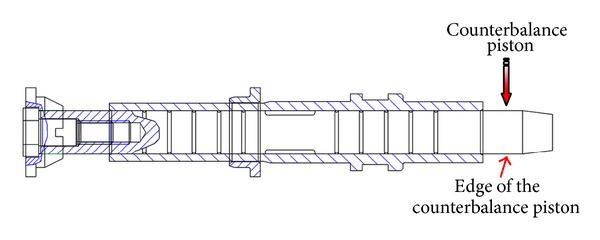
Sectional view of counterbalance assembly.

A schematic diagram of a swash plate VDAPP with a constant power regulator is shown in Figure 1.  Figure 2 represent hydraulic circuit of the constant power regulator system. The constant power regulator system consists of five parts, that is, a regulator assembly (A), a control cylinder assembly (B) which controls the angle of the swash plate, a counterbalance assembly (C), a swash plate (D), and a piston (E). As shown in Figure 3, the regulator assembly consists of a spool and sleeve. A flow area of the regulator system is determined by relative displacement between spool and sleeve. Figures 4 and 5 show the detailed structure of the control cylinder and counterbalance.

### 2.2. Operation of the Constant Power Regulator

**Figure 6:**
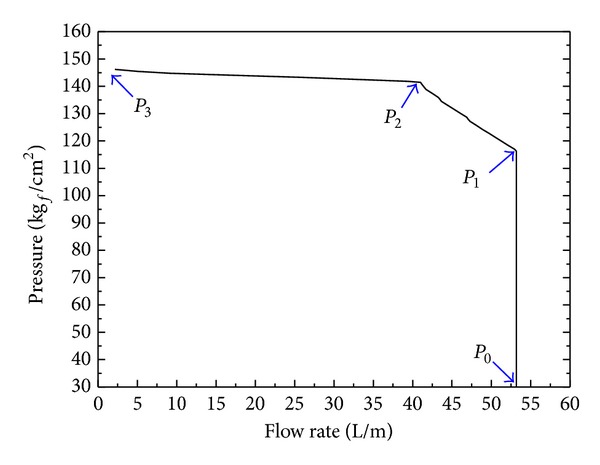
Examples of a desirable performance curve of constant power regulator system.

**Figure 7:**
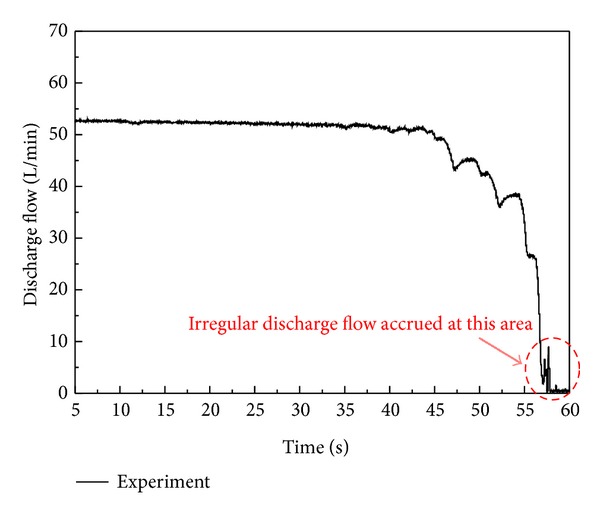
An irregular discharge flow characteristic at the cut-off pressure area of developed VDAPP in experiment.

Figure 6 shows desirable response of the constant power regulator system. As shown in Figure 7, on the other hand, the undesirable performance of an irregular discharge flow is observed at the cut-off pressure area in the performance test results of developed VDAPP. The maximum magnitude of the fluctuation is about 10 liters per minute as shown in Figure 7. In this study, therefore, the operation principle of the mechanical regulator with VDAPP is analyzed, and it is modeled by using AMESim software in order to find out the cause of the undesirable performance. Firstly, in this section, the operation principle of the mechanical regulator with VDAPP is described.

As shown in Figure 6, the output of the VDAPP with the constant power regulator is divided into three operating areas. That is maximum flow rate (*P*_0_ → *P*_1_), constant power (*P*_1_ → *P*_2_), and cut-off pressure (*P*_2_ → *P*_3_). These distinct characteristics are determined by the complex interaction of the regulator assembly, control cylinder assembly, and counterbalance assembly. The operating principle of these parts in each area is as follows.

#### 2.2.1. Maximum Flow Rate Area (*P*_0_ → *P*_1_)

**Figure 8:**
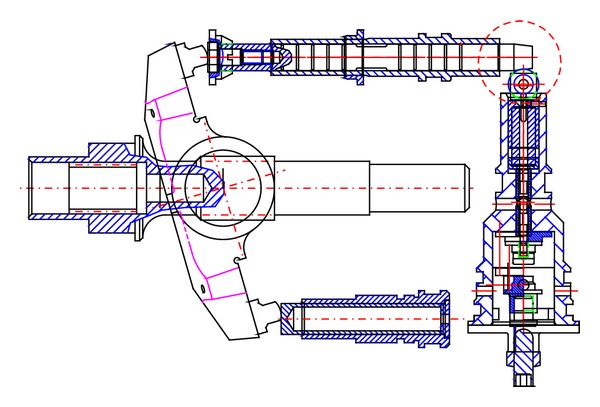
Schematic of VDAPP in the case of maximum flow rate.

**Figure 9:**
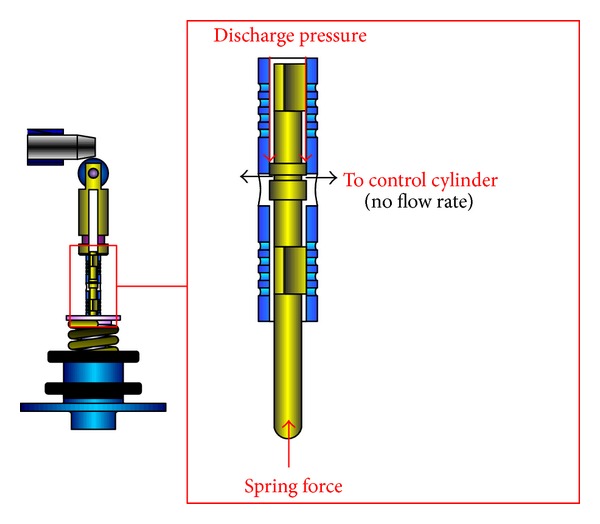
Schematic of constant power regulator in the case of maximum flow rate.

As shown in Figure 8, the swash plate is held in a certain swivel angle. In this area, the discharge pressure of the pump does not feed back into the control cylinder. This causes the swash plate to rotate in a maximum angular displacement. As a result, the pump can supply the maximum flow rate to a load system unless the discharge pressure of VDAPP is sufficiently increased to a certain level by a load. At the maximum flow rate section shown in Figure 9, the discharge flow rate cannot be feed into the control cylinder because the spool blocks the path of the sleeve.

#### 2.2.2. Constant Power Area (*P*_1_ → *P*_2_)

**Figure 10:**
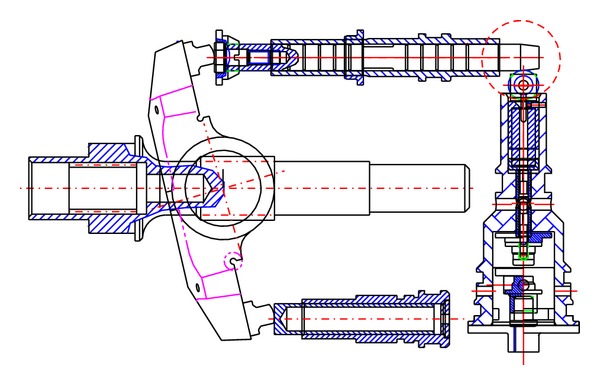
Schematic of VDAPP for the case of constant power.

The increased load pressure makes the spool move, and pilot flow rate is supplied into the control cylinder [7–9]. Then, the swivel angle is decreased as shown in Figure 10. By the kinematic constraints of the piston, the sleeve acts as a reaction force to the swivel torque. During this time, the swivel angle of the swash plate should be reduced gradually in order that the VDAPP can discharge the flow rate with constant power.

**Figure 11:**
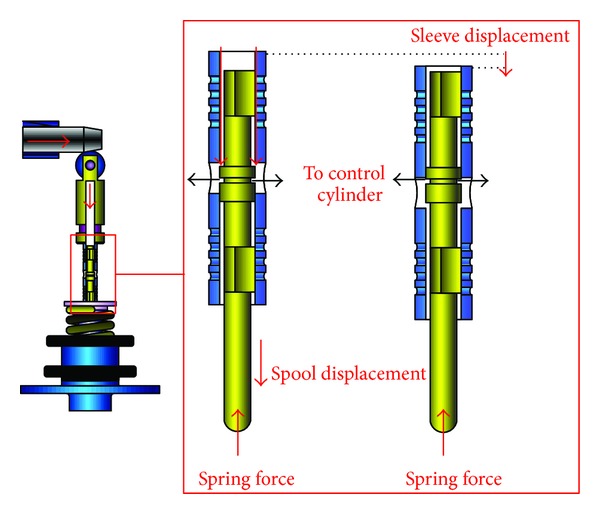
Schematic of constant power regulator for the case of constant power.

In the constant power area shown in Figure 11, the spool is moved by the pilot pressure which is equal to load pressure, and the spool displacement makes the flow path to the control cylinder open. Then, the flow is supplied to the control cylinder. Therefore, the swivel angle is decreased, and the discharge flow rate of the pump is reduced. When the swivel angle is decreased, the sleeve reduces or blocks the flow to the control cylinder by the movement of the counterbalance piston. Therefore, the displacement of the control cylinder is adjusted according to the load variation. Consequently, the increase of the load pressure decreases the discharge flow rate of VDAPP, and that makes output power of VDAPP constant because the output power of VDAPP is determined by the product of load pressure and discharge flow rate.

#### 2.2.3. Pressure Cut-off Area (*P*_2_ → *P*_3_)

**Figure 12:**
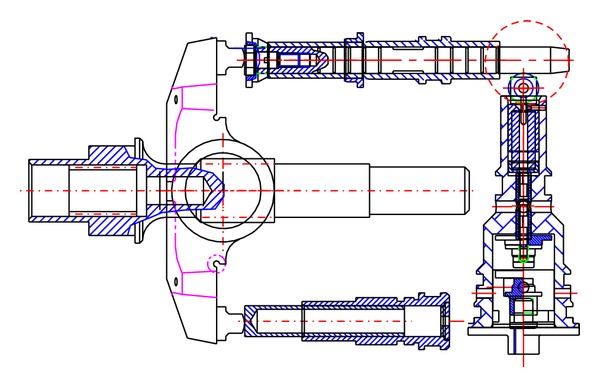
Schematic of VDAPP in the case of flow rate cut-off.

**Figure 13:**
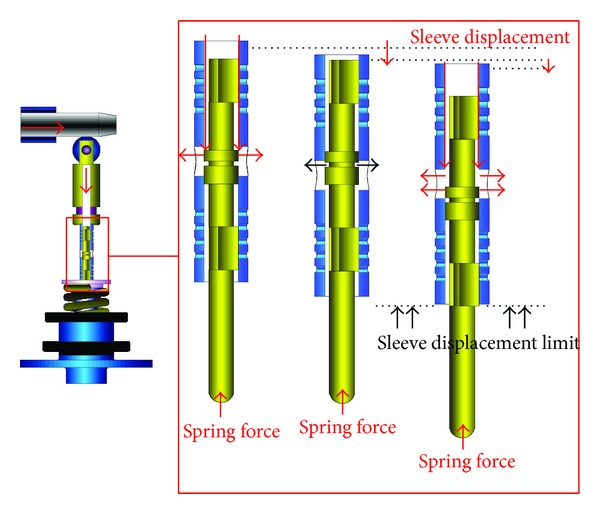
Schematic of constant power regulator in the case of flow rate cut-off.

When the load pressure reaches a limit, the VDAPP makes the discharge flow zero by setting the swivel angle of swash plate vertically, as shown in Figure 12. In this section, the discharge flow rate from VDAPP is rapidly decreased because the sleeve stroke is blocked by the kinematic constraint of the regulator. Then, the VDAPP sets the swash plate vertically and cuts off the discharge flow rate, as shown in Figure 13. 

As previously described, these characteristics of the pump-regulator assembly are determined by the interaction of the spool and sleeve. The pilot pressure generated by the load pressure of the system affects the spool.

## 3. Simulation Model of the Constant Power Regulator

In this study, the mechanical regulator with VDAPP is modeled by using AMESim software. Even though detailed design parameters can be considered in AMESim model, theoretical understandings of the mechanical regulator must be preceded in order to perform more accurate modeling by using AMESim. Therefore, the mathematical modeling of the mechanical regulator is deduced in this section.

### 3.1. Modeling the Constant Power Regulator

The spool moves when the force generated by the pilot pressure is greater than the reaction force due to the spring. The force balance acting on the spool can be represented as follows:

(1) pspAsp=mspx¨sp+bspxsp+ksp(x0sp+xsp)+Fspr+Fspf,

where *p*_sp_ is the pilot pressure, *A*_sp_ is the section area of the spool, *m*_sp_ is the mass of the spool, *b*_sp_ is the viscous friction coefficient between the spool and sleeve, *k*_sp_ is the reacting spring constant of the spool, *x*_0sp_ is the initial stroke, *x*_sp_ is the stroke, *F*_spr_ is the friction force of the spool, and *F*_spf_ = *ρQ* ^2^/*C*_*d*_ *A* is the flow force on the spool.

On the other hand, the stroke of the sleeve *x*_sl_ can be represented by the piston stroke of the counter balance *x*_cb_ as follows:

(2) xsl=x0sl+xcbtanθ,

where *x*_0sl_ is the displacement of the control cylinder at the operating point. 

In the VDAPP, the displacements of the control cylinder and the counterbalance piston are the same due to kinematic constraint. Therefore, (2) can be expressed as

(3) xsl=x0sl+xctanθ,

where *x*_*c*_ is the displacement of the control cylinder and *θ* is the taper angle of the counterbalance piston.

The force balance acting on the control cylinder can be represented as follows [10]:

(4) pcAc=mcx¨c+bcxc+kc(x0c+xc)+kslxslsinθcos⁡θ,

where *p*_*c*_ is the pressure in control cylinder, *A*_*c*_ is the pressurized area of the control cylinder, *m*_*c*_ is the mass of the control cylinder, *b*_*c*_ is the viscous friction coefficient between the control cylinder and a sleeve, *k*_*c*_ is the spring constant, *x*_0*c*_ is the initial displacement, *x*_*c*_ is the displacement of control cylinder, and *k*_sl_ is the spring constant of the reaction spring for the sleeve of the constant power mechanical regulator.

**Figure 14:**
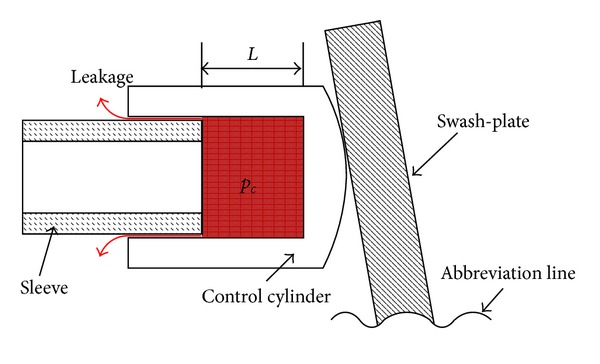
Schematic of the control cylinder [9].

Figure 14 shows a schematic of the control cylinder. The control pressure within the large servo chamber is governed by the pressure rise rate equation and is given by [9]

(5) p˙c=βAcx0c(Qin−Qout−Acx˙c−CLpc),

where *β* is the effective bulk modulus, *Q*_in_ is input flow rate to control cylinder, *Q*_out_ is output flow rate from control cylinder to reservoir, *A*_*c*_ is the pressurized area of control cylinder, and *C*_*L*_ is leakage coefficient of the control cylinder. At mechanical-type constant power regulators, the control flow varies according to the relative displacement between the spool and sleeve. Thus,

(6) xdiff=xsp−xsl.

If this relative difference *x*_diff_ larger than zero, the flow rate supply to the control cylinder. On the other hand, if *x*_diff_ is less than zero, the flow rate drains to the reservoir from the control cylinder. This can be expressed as

(7) Qin=CdAin(xdiff)2(pd−pc)ρ, Qout=0, (xdiff≥0),Qout=CdAout(xdiff)2pcρ, Qin=0, (xdiff<0),

where *C*_*d*_ is flow coefficient of orifice, *A*_in_(*x*_diff_) and *A*_out_(*x*_diff_) represent the orifice areas, *p*_*d*_ is discharge pressure of the hydraulic pump, and *ρ* is density of working fluid.

**Figure 15:**
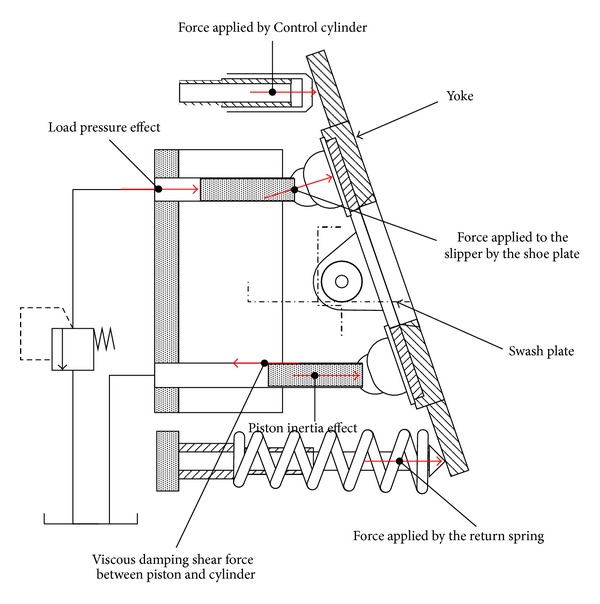
Various forces acting on the control cylinder [9, 12, 13].

The displacement of the control cylinder, *x*_*c*_ in (4), is determined by the resultant force on the swash plate as shown in Figure 15. The various forces are expressed in the form of a complex nonlinear model. In this study, in order to derive more accurate results, the VDAPP was also implemented using AMESim software.

### 3.2. Swashplate-Type VDAPP Model

**Figure 16:**
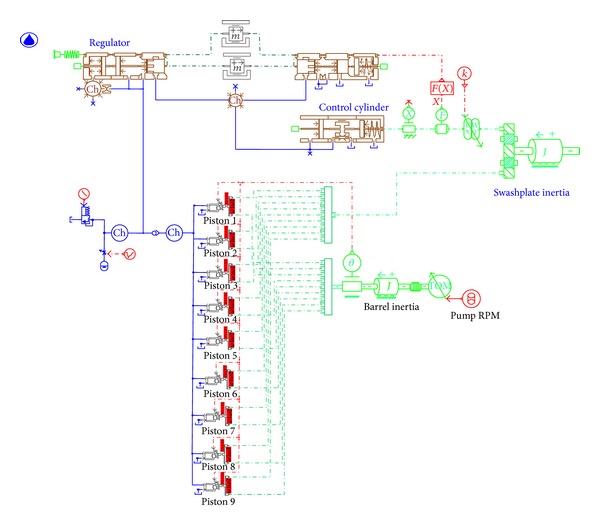
Simulation diagram of constant power regulator system based on AMESim software.

A VDAPP with a mechanical regulator system was established using AMESim simulation software, which allows a very accurate implementation of the response of a nonlinear system. In the field of hydraulic component design, AMESim is widely used to optimization and performance improvement as a review of the actual system [5].  Figure 16 shows an AMESim diagram for the analysis of the system performance of an MPR system that consists of nine pistons. 

The maximum swivel angle was set to 16°, which is the same as in the real component, and the exclusion volume was set to 11.6 cm^3^/rev. All parameters of the VDAPP are the actual design values used in the experimental equipment. The experimental equipment was modeled by considering the nonlinear behavior of the MPR pump system.

If the pump is composed of an odd number of pistons, the number of discharging pistons *z*_0_ is determined by the rotation angle of the piston, which located at regular intervals on the plate as follows [11]:

(8) z0=z+12,  (θ=0~πz),z0=z2,  (θ=πz~2πz).

**Figure 17:**
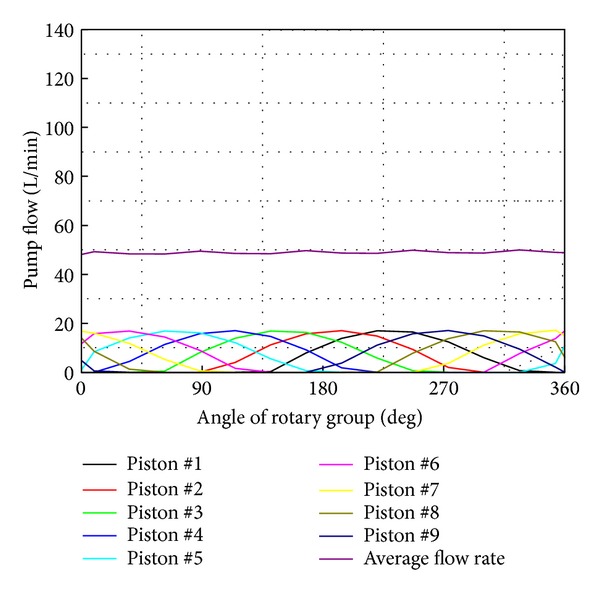
Simulation result of discharge flow ripple for variable displacement axial piston pump.

Figure 17 shows the simulation result when the pump is driven at 4500 rpm under no-load condition. The discharge flow rate is the sum of the flow rate of each piston. The pulsation in flow rate is observed in simulation result as shown in Figure 17. This simulation results also show that the average value of the discharge flow rate 49.8 L/min is less than the theoretical one 52 L/min because the internal leakage through the gap between the piston and cylinder block is considered in computer simulation. 

According to the results based on this simulation, the volumetric efficiency of the VDAPP is approximately 96%. A common value of volumetric efficiency of the VDAPP is 90 to 98%. Therefore, the VDAPP has satisfactory volumetric efficiency.

**Figure 18:**
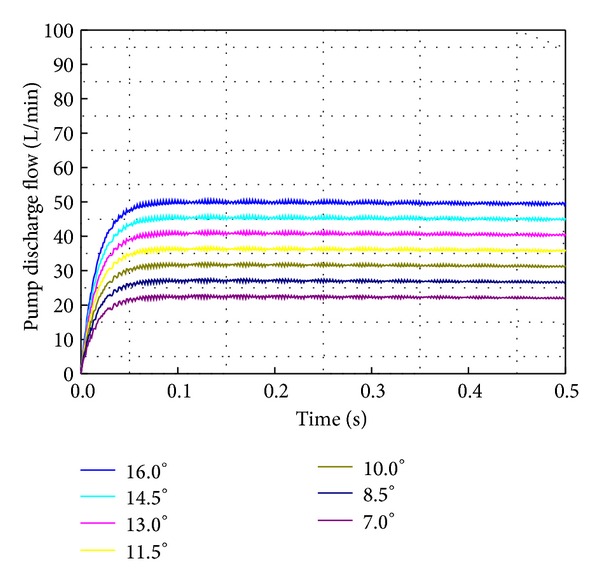
Simulation result of discharge flow ripple according to swash plate angle.

Figure 18 depicts the simulation result when the swivel angle changes from 7 to 16° at intervals of 1.5° to step 7 under no-load condition. This result shows that the discharge flow increases proportionally to the swivel angle. As shown in Figures 17 and 18, the AMESim simulation can implement the main characteristics of the VDAPP.

## 4. Analysis and Verification

In this section, problems of the mechanical regulator are considered and analyzed by AMESim simulation. Simulation results are compared with the experimental output of the real system in order to verify accuracy. In the AMESim simulation, all of the external conditions and internal parameters were set to be the same as those in the experiment.

**Figure 19:**
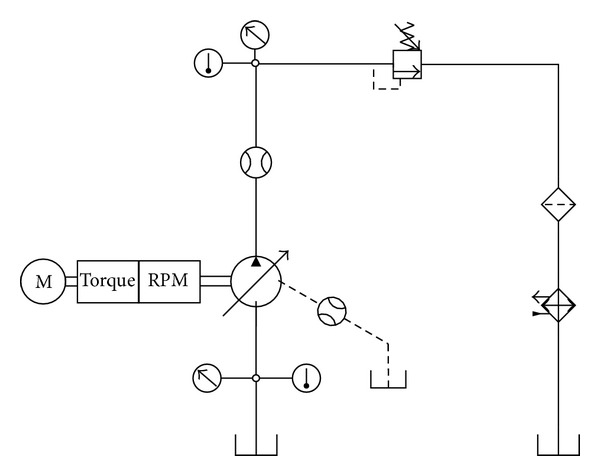
Hydraulic circiut for pump test rig.

Figure 19 shows the hydraulic circuit of test rig for VDAPP. The angular velocity of the electric motor is regulated as 4500 rpm, and the load pressure is adjusted by adjustable relief valve which installed in the discharge line of the VDAPP. The discharge pressure is slowly increased during 45 seconds. The load pressure, the discharge flow rate, and the angular velocity and the torque of the electric motor are acquired by data acquisition board in real time.

**Figure 20:**
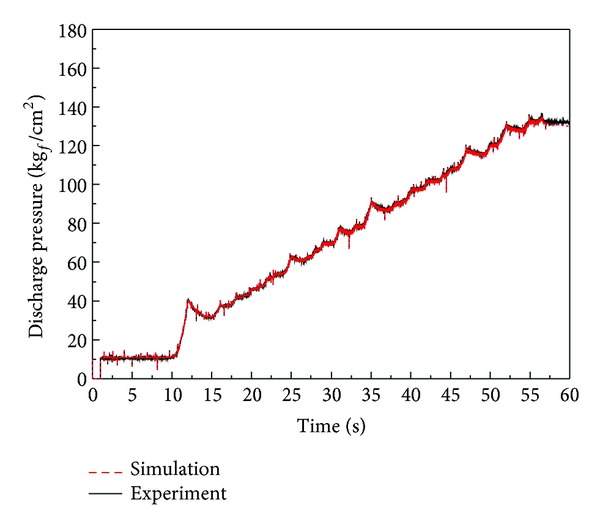
Condition of load pressure for constant power regulator system.

Figure 20 shows the load pressure regarding the reference input in this simulation. In order to implement the same load conditions, the output that was derived from the mechanical regulator with VDAPP was used in the AMESim simulation. Since the simulation operates under the same conditions as the experiment, the behavior of the regulator spool and sleeve can be inferred if the outputs of the experiment and simulation are similar.

**Figure 21:**
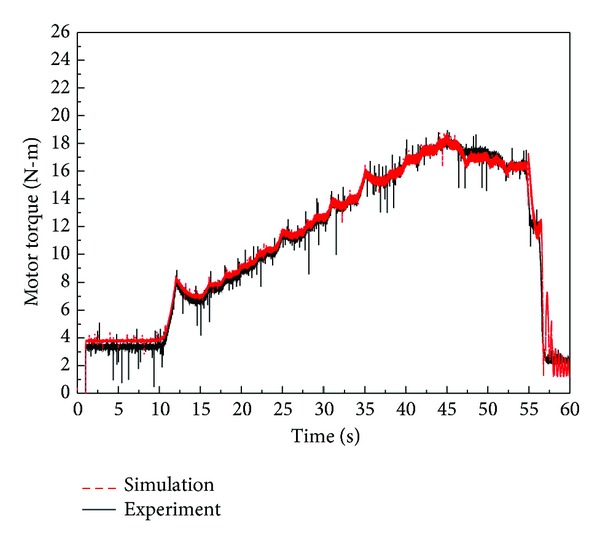
Simulation and experimental results for motor torque.

**Figure 22:**
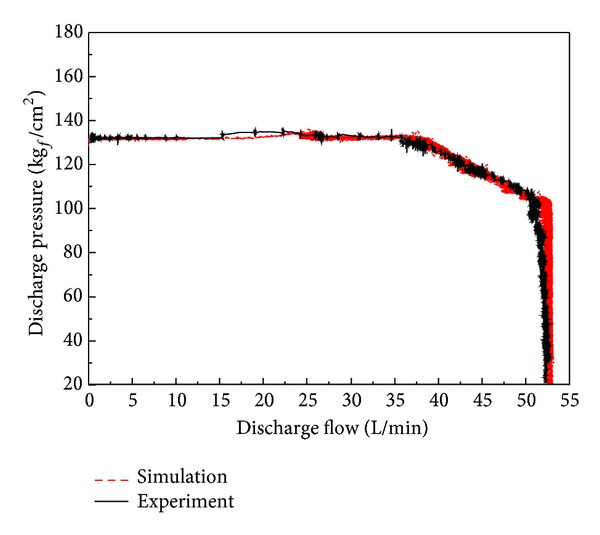
Comparison of flow-pressure curve based on simulation and experiment.

In Figure 21, we compare the results of the motor torque test.  Figure 22 shows the flow-pressure curves. The experimental output of the constant power test is also consistent with the simulation results. We found that the characteristics of the real component are well implemented through a comparison between simulation and experimental results.

**Figure 23:**
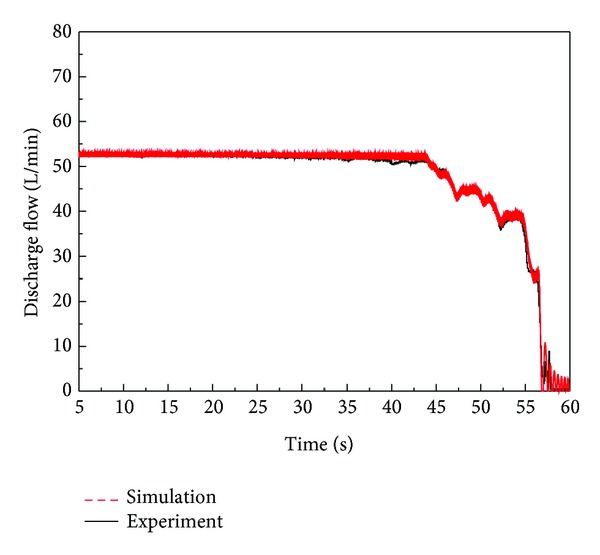
Output of discharge flow through simulation and experiment.

The discharge flow rate for this test is shown in Figure 23. In accordance with this result, irregular discharge flow was supplied to the system in the pressure cut-off area and the constant power area. An analysis of the internal motion of the spool and sleeve in the regulator, which cannot be measured in a real system, was performed by using AMESim software. As shown in Figure 12, the flow rate oscillates more frequently in the simulation results, but the maximum amplitude is in good agreement with the experimental results.

**Figure 24:**
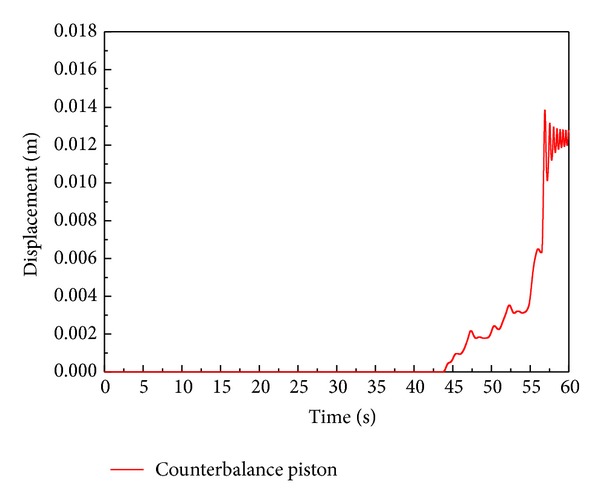
Simulation results of the displacement of the counterbalance piston.

Also, the displacement of the counterbalance piston in this simulation is shown in Figure 24. This result is in good agreement with the designed dimension of the real system.

On the other hand, an irregular fluctuation in displacement of the counterbalance piston causes pulsation of the discharge flow rate of the VDAPP. As discussed in previous section, the displacements of the control cylinder and the counterbalance piston are the same due to kinematic constraint. The pressures in the control cylinder affects to the displacement of it. The pressure in control cylinder is regulated by the balance of inlet/outlet flow rate in the volume of control cylinder. In addition, the inlet flow rate to the control cylinder is decided by the relative displacement between the spool and sleeve. Therefore, the discharge flow rate of the VDAPP is influenced by the relative displacement between the spool and sleeve in the constant power regulator.

**Figure 25:**
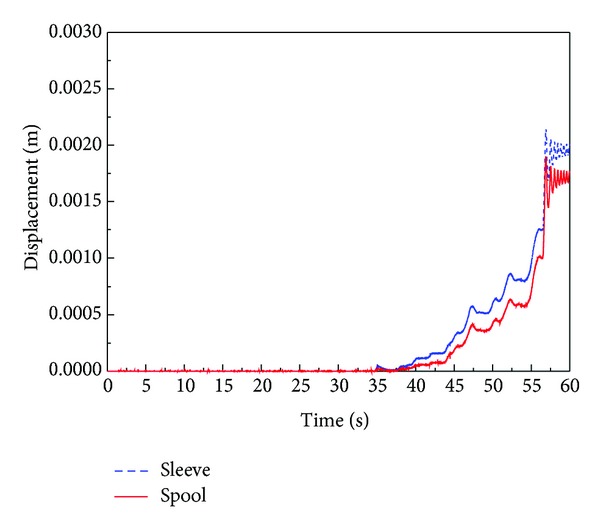
Simulation results of the displacement of the sleeve and spool of the regulator.

Figure 25 shows the simulation results of the displacements of the spool and sleeve versus time. The simulation results in Figure 24 show that the displacement of the sleeve and spool is distinguished at about 35 seconds. This means that the orifice is open on this point, but counterbalance piston does not move until 44 seconds. This phenomenon can be explained as follows. Though the pilot flow rate is supplied to the control cylinder at about 35 seconds, the amount of inlet flow rate is less than that of leakage from control cylinder. Therefore, the pressure in control cylinder does not rise. The relative displacement between spool and sleeve becomes sufficiently larger at around 44 seconds. At this time, the inlet flow rate is larger than the leakage from control cylinder. Therefore, the pressure in control cylinder is rising and the counterbalance piston starts to move and a constant output control begins. 

By the relative motion of the spool and sleeve, the orifice of the regulator increases proportionally to 56 seconds, and constant power is suspended at that time. After 56 seconds, the orifice area reaches the maximum, and the swivel angle is set to zero. In this case, the swivel angle rapidly decreases and is cut off because the spring force of the sleeve, which is acting on the counterbalance spool, disappears immediately by the kinematic constraints.

The fluctuation in displacement of the spool and sleeve remarkably appears in the pressure cut-off area from 56 to 58 seconds. At this period, the displacement of the counterbalance piston also oscillates, and the irregular discharge flow rate of the VDAPP is observed. This phenomenon seems to be accrued due to the discontinuous shape at the edge of the counterbalance piston because the reacting spring force of the sleeve acting on the counterbalance piston disappears immediately at this region.

**Figure 26:**
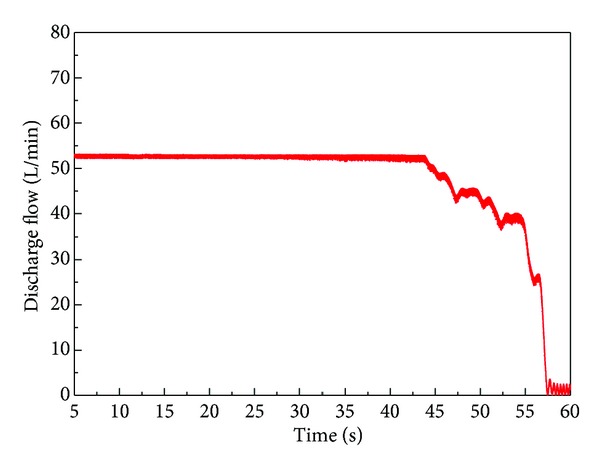
Output of discharge flow through simulation with modified counterbalance shape.

**Figure 27:**
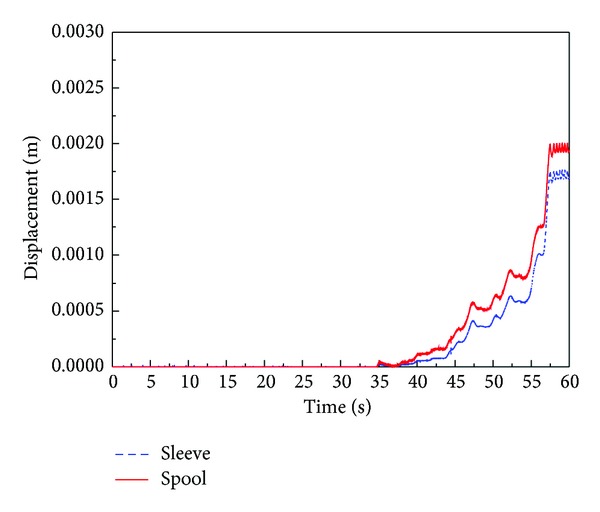
Simulation results of the displacement of the sleeve and spool of the regulator with modified counterbalance shape.

Therefore, we proposed the rounded shape for the edge of the counterbalance piston. The effect of rounded edge is implemented and verified by computer simulation based on the verified AMESim simulation model.  Figure 26 shows the simulation results of discharge flow rate in which improved shape of the edge of the counterbalance piston is adopted. Also, the behaviors of the spool and sleeve are presented in Figure 27. As shown in Figures 26 and 27, the maximum amplitude of oscillation of the spool and sleeve is reduced to half, and the irregular discharge flow rate is improved.

## 5. Conclusion

In this study, the constant power mechanical regulator system with variable displacement axial piston pump is considered. The constant power mechanical regulator with VDAPP has a problem of pulsation in the discharge flow rate at the cut-off area. In order to solve the problem, the internal behavior of the constant power regulator with VDAPP is analyzed by modeling the system using the AMESim software. The theoretical analysis of constant power regulator is induced for precise modeling, and the internal dynamics of un-measurable components are studied. The validation of the simulation model is confirmed by comparing the simulation results with the experimental output of the real system. By analyzing the dynamics of the unmeasurable internal components, it is found that the irregular discharge flow rate is caused by the discontinuous shape at the edge of the counterbalance piston. Therefore, we proposed the rounded shape for the edge of the counterbalance piston. The effect of the redesigned shape is implemented by AMESim simulation, and the validation is verified by computer simulation. The future work is experimental confirmation of the redesigned shape.
